# The E3 Ligase PIAS1 Regulates p53 Sumoylation to Control Stress-Induced Apoptosis of Lens Epithelial Cells Through the Proapoptotic Regulator Bax

**DOI:** 10.3389/fcell.2021.660494

**Published:** 2021-06-14

**Authors:** Qian Nie, Huimin Chen, Ming Zou, Ling Wang, Min Hou, Jia-Wen Xiang, Zhongwen Luo, Xiao-Dong Gong, Jia-Ling Fu, Yan Wang, Shu-Yu Zheng, Yuan Xiao, Yu-Wen Gan, Qian Gao, Yue-Yue Bai, Jing-Miao Wang, Lan Zhang, Xiang-Cheng Tang, Xuebin Hu, Lili Gong, Yizhi Liu, David Wan-Cheng Li

**Affiliations:** State Key Laboratory of Ophthalmology, Zhongshan Ophthalmic Center, Sun Yat-sen University, Guangzhou, China

**Keywords:** sumoylation, apoptosis, PIAS1, p53, Bax, lens, cataract, oxidative stress

## Abstract

Protein sumoylation is one of the most important post-translational modifications regulating many biological processes (Flotho A & Melchior F. 2013. *Ann Rev. Biochem*. 82:357–85). Our previous studies have shown that sumoylation plays a fundamental role in regulating lens differentiation (Yan et al., 2010. *PNAS*, 107(49):21034-9.; Gong et al., 2014. *PNAS*. 111(15):5574–9). Whether sumoylation is implicated in lens pathogenesis remains elusive. Here, we present evidence to show that the protein inhibitor of activated STAT-1 (PIAS1), a E3 ligase for sumoylation, is implicated in regulating stress-induced lens pathogenesis. During oxidative stress-induced cataractogenesis, expression of PIAS1 is significantly altered at both mRNA and protein levels. Upregulation and overexpression of exogenous PIAS1 significantly enhances stress-induced apoptosis. In contrast, silence of PIAS1 with CRISPR/Cas9 technology attenuates stress-induced apoptosis. Mechanistically, different from other cells, PIAS1 has little effect to activate JNK but upregulates Bax, a major proapoptotic regulator. Moreover, Bax upregulation is derived from the enhanced transcription activity of the upstream transcription factor, p53. As revealed previously in other cells by different laboratories, our data also demonstrate that PIAS1 promotes SUMO1 conjugation of p53 at K386 residue in lens epithelial cells and thus enhances p53 transcription activity to promote Bax upregulation. Silence of Bax expression largely abrogates PIAS1-mediated enhancement of stress-induced apoptosis. Thus, our results demonstrated that PIAS1 promotes oxidative stress-induced apoptosis through positive control of p53, which specifically upregulates expression of the downstream proapoptotic regulator Bax. As a result, PIAS1-promoted apoptosis induced by oxidative stress is implicated in lens pathogenesis.

## Introduction

Sumoylation is a unique class of protein post-translational modification, discovered in the past century (Boddy et al., 1996; Matunis et al., 1996; Okura et al., 1996; Shen et al., 1996). The small ubiquitin-like modifier (SUMO) proteins can interact with over 6,000 proteins, regulating their activity, interactions, localization, or stability through a reversible covalent modification (Geiss-Friedlander and Melchior, 2007; Flotho and Melchior, 2013; Sheng et al., 2019). Three major SUMO isoforms have been identified in vertebrates, which are known as SUMO1, SUMO2, and SUMO3 (Hay, 2005).

Sumoylation is an enzymatic reaction catalyzed by the E1 activating enzyme, the E2 conjugating enzyme, and several E3 ligases in the presence of ATP as energy supply, and reversed by several SUMO isopeptidases known as SENPs (Johnson, 2004; Hickey et al., 2012). Previous studies have shown that protein sumoylation plays an important role in regulating different biological processes including cell division, autophagy, transformation, aging, and apoptosis (Johnson, 2004; Hendriks and Vertegaal, 2016). Moreover, sumoylation is also implicated in various human diseases caused by gene mutation and stress response (Flotho and Melchior, 2013; Seeler and Dejean, 2017; Yang et al., 2017; Sheng et al., 2019; Princz and Tavernarakis, 2020).

During sumoylation reaction, E3 ligases have been reported to stabilize the binding between the SUMO target proteins and the E2 conjugating enzyme, controlling the transfer of SUMO from E2 to substrate proteins (Hay, 2005; Hickey et al., 2012; Flotho and Melchior, 2013). Since the E1 activating enzyme (consisting of SAE1-UBA2 heterodimer) and the E2 conjugating enzyme (UBC9) are the unique enzymes, the E3 ligases play a key role in the selection of SUMO isoforms and target proteins (Hay, 2005; Geiss-Friedlander and Melchior, 2007; Hendriks and Vertegaal, 2016; Pichler et al., 2017). In mammals, the protein inhibitor of activated STAT (PIAS) family of E3 ligases contain five subtypes, namely, PIAS1, PIAS2a, PIAS2b, PIAS3, and PIAS4, among which PIAS1 is known to promote cell apoptosis depending on its SUMO E3 ligase activity (Shuai and Liu, 2005; Palvimo, 2007; Rytinki et al., 2009; Sudharsan and Azuma, 2012; Yang et al., 2013; Rabellino et al., 2017; Li et al., 2020). PIAS1 can regulate apoptosis by mediating sumoylation of both transcription factors and kinases (Liu and Shuai, 2001; Megidish et al., 2002; Shishido et al., 2008; Alm-Kristiansen et al., 2011; Leitao et al., 2011; Sudharsan and Azuma, 2012; Li et al., 2013; Yang et al., 2013; Chiou et al., 2014; Wang et al., 2018) and thus becomes involved in various human diseases including cardiovascular diseases, neuronal disorder, and cancer development (Fatkin et al., 1999; Sternsdorf et al., 1999; Li et al., 2003; Steffan et al., 2004; Kim et al., 2006, 2007; Ballatore et al., 2007; Evdokimov et al., 2008; Subramaniam et al., 2009; Flotho and Melchior, 2013; Yang et al., 2017). One of the targets modified by PIAS1 is the tumor suppressor p53. Several laboratories have shown that p53 can be conjugated by SUMO1 through the action of PIAS1 (Gostissa et al., 1999; Rodriguez et al., 1999; Muller et al., 2000; Kahyo et al., 2001; Kwek et al., 2001; Schmidt and Muller, 2002; Okubo et al., 2004). Whether PIAS1 is implicated in ocular diseases, especially lens cataractogenesis, remains elusive.

Cataract is an aging disease that, in most cases, is derived from aging process or stress induction such as oxidative stress (Spector, 1995 and references therein). Mechanistically, we have previously demonstrated that stress-induced apoptosis is a common cellular basis for non-congenital cataractogenesis (Li et al., 1995a,b; Li and Spector, 1996). In lens epithelial cells (LECs), apoptosis is mainly mediated by endogenous and exogenous apoptotic pathways (Li, 1997; Li et al., 2001; Mao et al., 2001, 2004; Yao et al., 2003; Kim and Koh, 2011). Bcl-2 family proteins play a crucial role in mediating endogenous apoptotic pathway, and among which Bax is one of the most important pro-apoptotic proteins (Mao et al., 2004; Czabotar et al., 2014; Singh et al., 2019; Walensky, 2019; Moldoveanu and Czabotar, 2020).

In this study, we present evidence to show that during oxidative stress-induced apoptosis, PIAS1 expression is significantly changed at the mRNA and protein levels. The change in PIAS1 expression is closely related with apoptosis. PIAS1 knockout *via* CRISPR/Cas9 technology attenuates oxidative stress-induced apoptosis. In contrast, expression of exogenous PIAS1 enhances oxidative stress-induced apoptosis of LECs. To define the underlying mechanism, we analyzed the apoptosis-related factors in PIAS1 overexpression and knockout cells. Our results reveal that PIAS1 affects the expression level of the pro-apoptotic protein Bax. Furthermore, we demonstrate that PIAS1 regulation of Bax occurs through regulation of p53 sumoylation at the K386 residue in LECs. The sumoylation-deficient p53 K386R mutant can protect cells against the stress-induced apoptosis. Finally, in Bax knockout cells, we found that absence of Bax significantly abrogates PIAS1-mediated apoptosis under oxidative stress induction. Taken together, our results demonstrate that PIAS1 is implicated in lens cataractogenesis. Mechanistically, PIAS1 sumoylates p53 at K386 to upregulate Bax and thus promotes oxidative stress-induced apoptosis through p53-Bax pathway.

## Results

### Oxidative Stress Induces PIAS1 Alteration in Lens Epithelial Cells

**FIGURE 1:**
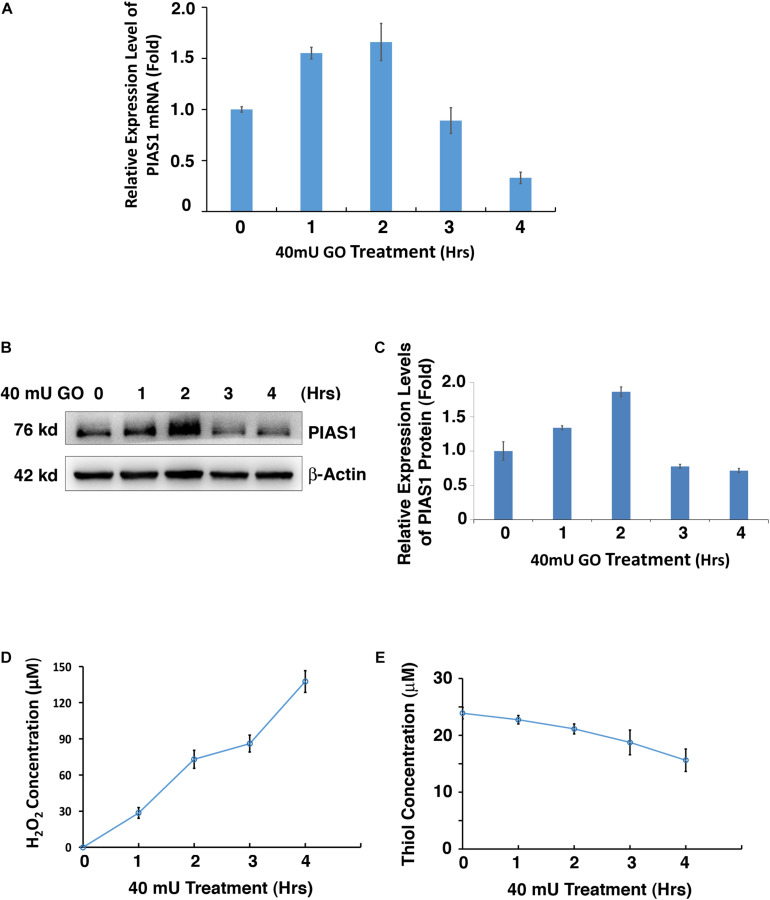
Oxidative stress regulates PIAS1 expression in lens epithelial cells. **(A)** The expression of mRNA levels of PIAS1 under 40 mU GO treatment from 0 to 4 h was determined by real-time PCR. Ct values of each sample were normalized with the Ct value of β-actin. **(B)** Western blot analysis of PIAS1 protein level under 40 mU GO treatment from 0 to 4 h. The β-actin was used as a loading control. **(C)** Quantification of the Western blot results in panel **(A)**. **(D)** Dynamic H_2_O_2_ concentration generated from 40 mU GO in αTN4-1 cells from 0 to 4 h. **(E)** Dynamic changes of free thiol content upon 40 mU GO treatment in αTN4-1 cells from 0 to 4 h. All experiments were repeated three times. Error bar represents standard deviation, *N* = 3.

It is well established that oxidative stress plays a causing role in cataractogenesis (Giblin et al., 1995; Li et al., 1995a,b; Spector, 1995; Li and Spector, 1996; Reddy et al., 2001; Raghavan et al., 2016; Rakete and Nagaraj, 2016; Barnes and Quinlan, 2017; Fan et al., 2017; Wang et al., 2017). In our glucose oxidase (GO) treatment-induced cataract model (Supplementary Figure 1), we found that GO also regulates PIAS1 expression. As shown in Figure 1A, 40 mU GO induced time-dependent upregulation of PIAS1 mRNA in the first 2 h. As treatment time was extended, PIAS1 mRNA level became downregulated. Similar pattern of PIAS1 protein expression was observed (Figures 1B,C). As GO concentration was increased, PIAS1 expression was downregulated (Supplementary Figure 2A). Consistent with our previous studies (Sun et al., 2020; Wang et al., 2020), GO treatment generated hydrogen peroxide (Figure 1D and Supplementary Figure 2C) and caused a significant drop of the free thiol level (Figure 1E and Supplementary Figure 2D). These results indicate that PIAS1 is regulated by oxidative stress. Whether the change of PIAS1 level is linked to lens pathology remains to be further studied.

### PIAS1 Promotes Oxidative Stress-Induced Apoptosis of Lens Epithelial Cells

**FIGURE 2:**
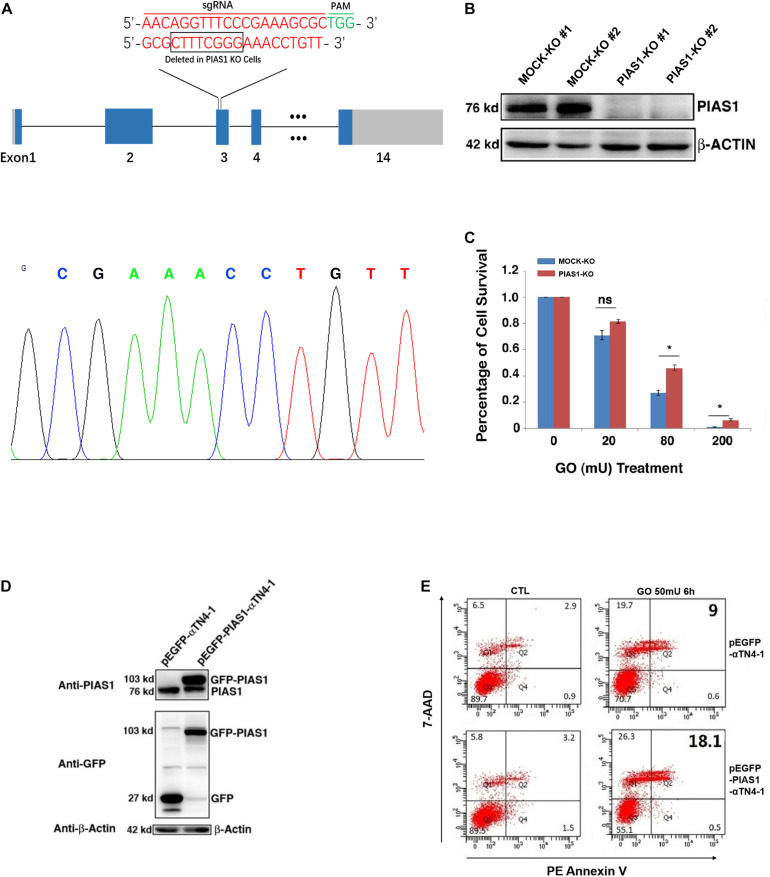
PIAS1 promotes oxidative stress-induced apoptosis of lens epithelial cells. **(A)** Schematic diagram of the strategy for generating the PIAS1 knockout cells by CRISPR/Cas9 gene editing technology. Two sets of sgRNAs were used to generate the knockout cell line (top). Eight nucleotides were deleted in exon 3 and the deletion was verified by DNA sequencing (bottom). The mapping sequence of the genomic DNA with deletion in PIAS1 knockout cells was shown. PAM, protospacer adjacent motif. **(B)** Western blot analysis of the expression levels of PIAS1 in αTN4-1 negative-control vector (MOCK-KO) and PIAS1 knockout (PIAS1-KO) cells. Note that expression of PIAS1 was not detectable in PIAS1 knockout cells. The β-actin was used as a loading control. **(C)** Apoptosis rate in MOCK-KO ad PIAS1-KO cells under treatment of 20, 80, and 200 mU GO for 3 h as measured with CellTiter-Glo^®^ Luminescent Cell Viability Assay analysis. **(D)** Western blot analysis of endogenous and exogenous expression of PIAS1 in pEGFP-αTN4-1 and pEGFP-PIAS1-αTN4-1 cells detected with anti-GFP and anti-PIAS1 antibodies. The β-actin was used as a loading control. **(E)** Apoptosis rate in pEGFP-αTN4-1 and pEGFP-PIAS1-αTN4-1 cells under treatment of 50 mU GO for 6 h measured by flow cytometry. Cells with the indicated treatment were stained with phycoerythrin annexin V (PE) and 7-amino-actinomycin (7-ADD) before flow cytometry analysis. Numbers indicate percentage of apoptotic and non-apoptotic cells in each gate. All experiments were repeated three times. Error bar represents standard deviation, *N* = 3. *p < 0.05; ns, statistically not significant.

Next, we test if GO-regulated changes in PIAS1 expression are linked to lens pathogenesis. Using CRISPR/Cas9 technology, we generated a PIAS1-knockout (KO) cell line with mouse lens epithelial cells, αTN4-1. The PIAS1 knockout strategy, as shown in Figure 2A, is conducted with the deletion of nucleotides in exon 3. The knockout result was confirmed by direct DNA sequencing and the absence of PIAS1 protein expression as verified by Western blot analysis (Figure 2B). Treatment of mock KO αTN4-1 cells (MOCK-KO) and PIAS1 KO cells with 20–200 mU GO for 3 h revealed differential apoptosis in the two types of cells. As shown in Figure 2C, cells with PIAS1 knockout displayed enhanced viability as measured by ATP loss. Next, we overexpressed PIAS1 by establishing pEGFP-C3-PIAS1 stable cell line with the vector pEGFP-C3 as control in αTN4-1 cells. The expression of EGFP or EGFP-PIAS1 fusion protein was verified by Western blot analysis using antibodies against PIAS1 and GFP (Figure 2D). We then performed flow cytometry analysis to detect possible differential apoptosis by staining with phycoerythrin annexin V (PE) and 7-amino-actinomycin (7-AAD) in EGFP expression and EGFP-PIAS1 fusion protein overexpression αTN4-1 cells. Compared with EGFP expression cells, overexpression of EGFP-PIAS1 displayed prominent sensitivity to oxidative stress by twofold under GO treatment (Figure 2E). Taken together, these results demonstrate that PIAS1 promotes oxidative stress-induced apoptosis of lens epithelial cells.

### PIAS1 Mediates p53 Sumoylation in Lens Epithelial Cells at the K386 Residue

**FIGURE 3:**
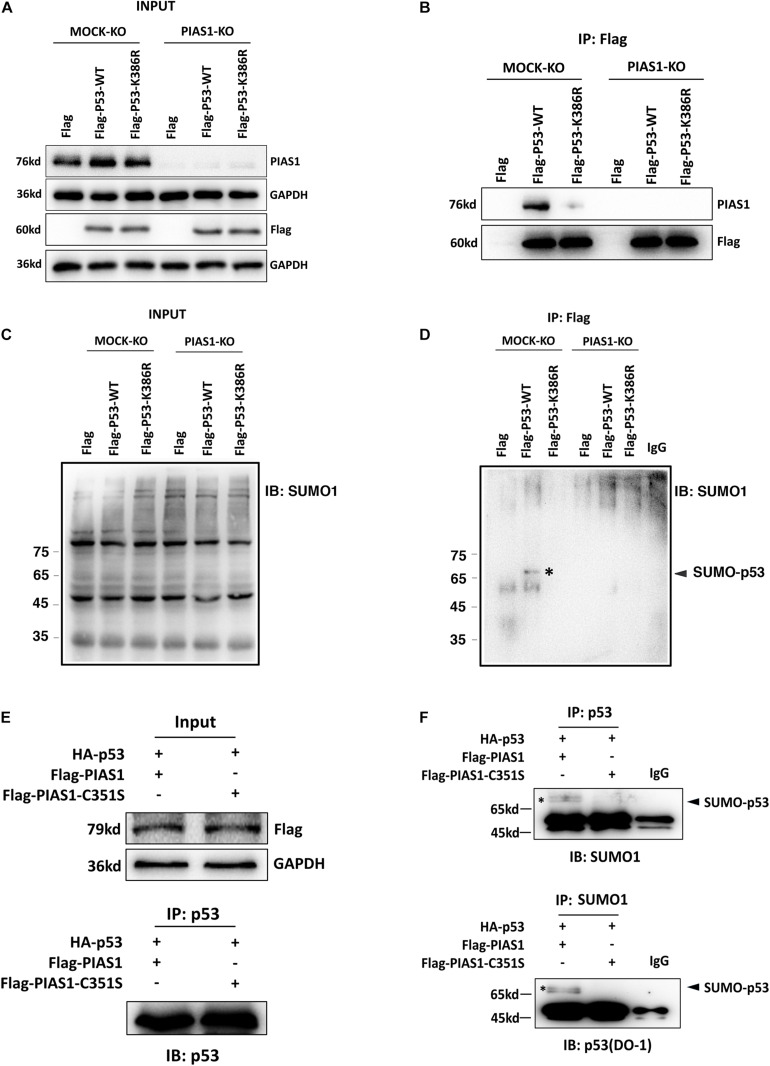
PIAS1 mediates p53 sumoylation in lens epithelial cells at the K386 residue. **(A–D)** MOCK-KO and PIAS1-KO cells were transfected with Flag, Flag-p53-WT, or Flag-p53-K386R as indicated. Forty-eight hours after transfection, whole-cell lysates were blotted (IB) with anti-PIAS1, anti-Flag antibodies **(A)**, and anti-SUMO1 antibody **(C)**. The cell lysates were immunoprecipitated (IP) with anti-Flag antibody, followed by blotting (IB) with anti-PIAS1, anti-Flag antibodies **(B)**, and anti-SUMO1 antibody **(D)**. The SUMO1-p53 conjugated band was detected (labeled with ^∗^). **(E,F)** MOCK-KO and PIAS1-KO cells were co-transfected with HA-p53 and Flag-PIAS1 or Flag-PIAS1-C351S plasmids as indicated. Forty-eight hours after transfection, whole-cell lysates were blotted (IB) with anti-Flag antibody **(E**, top). The cell lysates were immunoprecipitated (IP) with anti-p53 antibody, followed by blotting (IB) with anti-p53 (DO-1) antibody **(E**, bottom) and anti-SUMO1 antibody **(F**, top). In turn, the cell lysates were immunoprecipitated (IP) with anti-SUMO1 antibody, followed by blotting (IB) with anti-p53 (DO-1) antibody **(F**, bottom). The sumoylated p53 was detected (labeled with^∗^). The IP experiments were conducted in the presence of a desumoylation inhibitor, 20 mM NEM.

To address the underlying mechanism through which PIAS1 promotes oxidative stress-induced apoptosis, we first examined if PIAS1 can activate JNK kinases since previous studies have shown that PIAS1 can activate JNK to trigger apoptosis in different human cell lines (Liu and Shuai, 2001; Leitao et al., 2011). As shown in Supplementary Figure 3, PIAS1 overexpression or knockout did not change JNK1/2 total protein levels or activity levels (as reflected by JNK1/2 phosphorylation). Next, we examined if PIAS1 can act as a sumoylation E3 ligase to promote apoptosis. In this regard, since the tumor suppressor p53 is a master regulator of apoptosis (Kruiswijk et al., 2015) and early studies from numerous laboratories have shown that PIAS1 promotes SUMO1-conjugated sumoylation of p53 at K386 residue (Gostissa et al., 1999; Rodriguez et al., 1999; Muller et al., 2000; Kahyo et al., 2001; Kwek et al., 2001; Schmidt and Muller, 2002; Okubo et al., 2004), we explored if PIAS1 can also mediate p53 sumoylation in mouse lens epithelial cells, αTN4-1. First, immunoprecipitation (Co-IP) linked Western blot analysis with both anti-p53 and anti-SUMO1 antibodies revealed the presence of a band above the p53, which can be detected by both anti-p53 and anti-SUMO1 antibodies (Supplementary Figure 4). To confirm that PIAS1 indeed mediates p53 sumoylation in lens epithelial cells, we generated a K386R mutant p53 to disrupt its sumoylation. Both wild-type and K386R mutant p53 as well as the vector Flag were transfected into αTN4-1 cells without or with PIAS1 knockout. Co-IP analysis of the total proteins extracted from these different cell lines revealed the interaction between PIAS1 and the wild-type p53 but not the K386R-p53 (Figure 3B), and the presence of sumoylated p53 in Flag-p53-WT transfected cells but not in Flag-p53-K386R-transfected cells (Figure 3D). Moreover, sumoylated p53 was only detectable when wild-type PIAS1 was present (Figure 3F). When PIAS1 was mutated into Flag-PIAS1-C351S, the sumoylated p53 was no longer detectable (lane 2 of Figure 3F). Together, these results demonstrated that in lens epithelial cells, PIAS1 can mediate p53 sumoylation at K386 residue as previously detected in other cells (Gostissa et al., 1999; Rodriguez et al., 1999; Muller et al., 2000; Kahyo et al., 2001; Kwek et al., 2001; Schmidt and Muller, 2002; Okubo et al., 2004).

### PIAS1 Regulates Expression of the Pro-apoptotic Protein Bax in Lens Epithelial Cells

**FIGURE 4:**
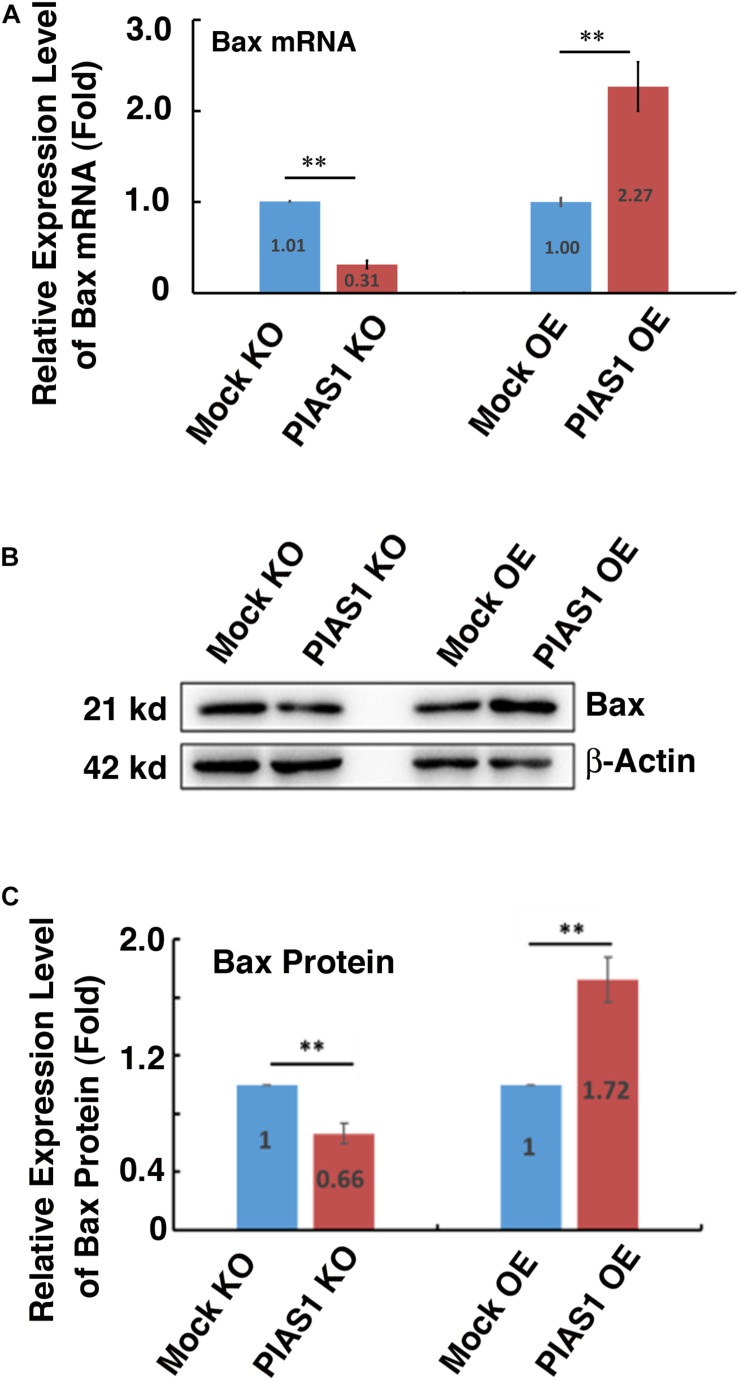
PIAS1 regulates expression of the pro-apoptotic protein Bax in lens epithelial cells. **(A)** qRT-PCR analysis of the mRNA expression level of Bax in MOCK-KO, PIAS1-KO, MOCK OE (pEGFP-αTN4-1), and PIAS1-OE (pEGFP-PIAS1-αTN4-1) cells. Cp values of each sample were normalized with the Cp value of β-actin. **(B)** Western blot analysis of the protein expression level of Bax in MOCK-KO and PIAS1-KO, MOCK OE (pEGFP-αTN4-1), and PIAS1-OE (pEGFP-PIAS1-αTN4-1) cells. The β-actin was used as a loading control. **(C)** Quantification of the Western blot results in panel **(B)**. All experiments were repeated three times. Error bar represents standard deviation, *N* = 3. ^∗∗^*p* < 0.01.

To further understand how PIAS1 promotes apoptosis of lens epithelial cells, we next examined the possible regulation of the Bcl-2 family members by PIAS1. As shown in Figure 4, the expression levels of the mRNA and protein for the proapoptotic regulator Bax were significantly downregulated in PIAS1 knockout cells. In contrast, the expression level of the protein for another proapoptotic regulator, Bak, remain almost unchanged (Supplementary Figure 5). Next, we examined whether overexpression of PIAS1 could regulate the expression level of Bax. As shown in Figure 4, Bax was significantly upregulated in PIAS1 overexpression cells. Thus, in lens epithelial cells, PIAS1 promotes apoptosis through regulation of Bax, a component of the intrinsic apoptotic pathway.

### Desumoylation of p53 Protects Lens Epithelial Cells From Oxidative Stress-Induced Apoptosis

**FIGURE 5:**
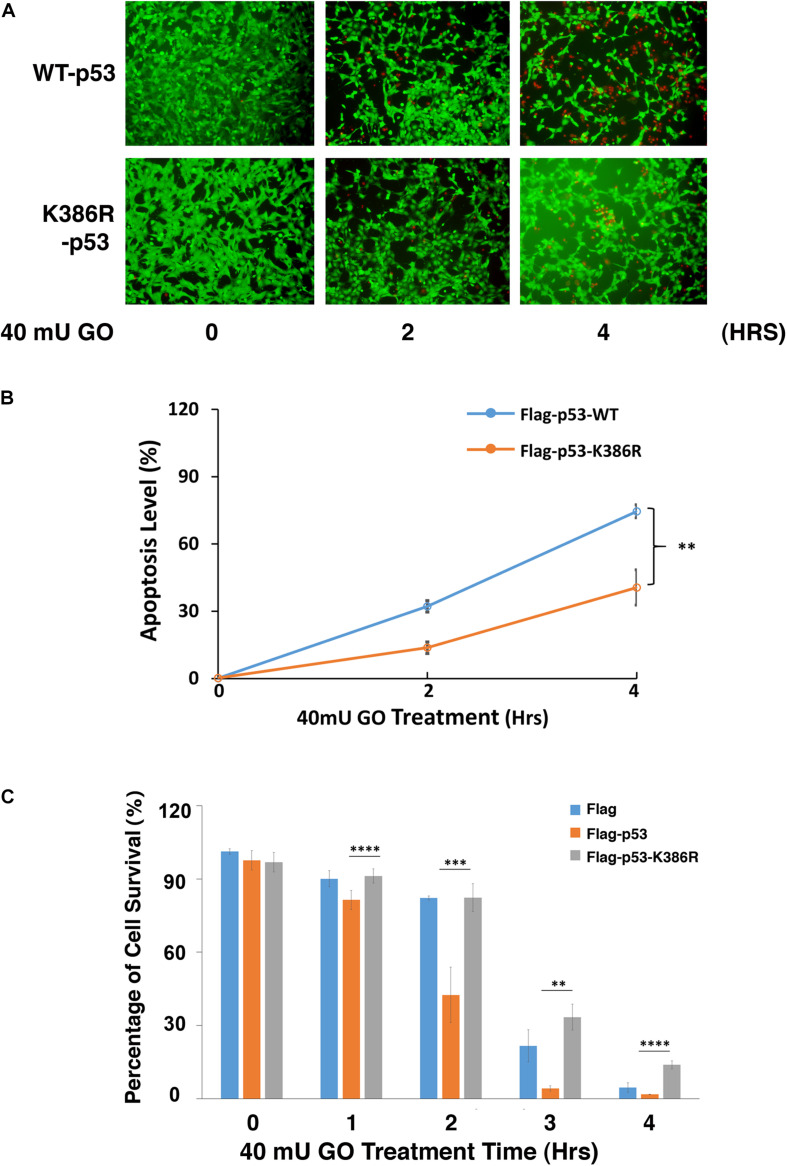
Desumoylation of p53 protects lens epithelial cells from oxidative stress-induced apoptosis. p53-KO αTN4-1 cells were transfected with Flag, Flag-p53-WT, or Flag-p53-K386R as indicated. **(A)** Forty-eight hours after transfection, Live/Dead Viability/Cytotoxicity assay was conducted to analyze cell apoptosis under 40 mU GO treatment for 0 to 4 h. **(B)** The live/dead cells in panel **(A)** were quantified from three different experiments. **(C)** The cellTiter-Glo^®^ luminescent cell viability assay was conducted to analyze cell apoptosis under 40 mU GO treatment for 0 to 4 h. All experiments were repeated three times. Error bar represents standard deviation, *N* = 3. ^∗∗^*p* < 0.01, ^∗∗∗^*p* < 0.005, ^*⁣*⁣**^p < 0.0001.

The positive regulation of PIAS1 on p53 sumoylation promotes us to further analyze the association between p53 sumoylation and oxidative stress-induced apoptosis in LECs. To do so, we first generated the p53 knockout cell line using CRISPR/Cas9 technology (Supplementary Figure 6). Then, we transfected the p53(-/-) αTN4-1 cells with Flag, Flag-p53-WT, and Flag-p53-K386R, respectively. Forty-eight hours post-transfection, the cells were treated with 40 mU GO and the apoptosis rate was examined using two methods: live/dead viability/cytotoxicity assay and ATP loss analysis. As shown in Figures 5A,B, Flag-p53-K386R-transfected cells were much more resistant against 40 mU GO-induced apoptosis than the Flag-p53-WT-transfected cells did. ATP loss analysis further confirmed that de-sumoylated p53 conferred much stronger resistance to oxidative stress-induced apoptosis than p53-WT did (Figure 5C). Interestingly, cells transfected with the Flag vector without p53 were also more sensitive to oxidative stress-induced apoptosis than the p53-K386R-transfected cells. Together, these results confirmed that PIAS1-mediated p53 sumoylation is implicated in oxidative stress-induced apoptosis of lens epithelial cells.

### PIAS1-Mediated p53 Sumoylation at K386 Enhances Expression of the Downstream Proapoptotic Regulator Bax

**FIGURE 6:**
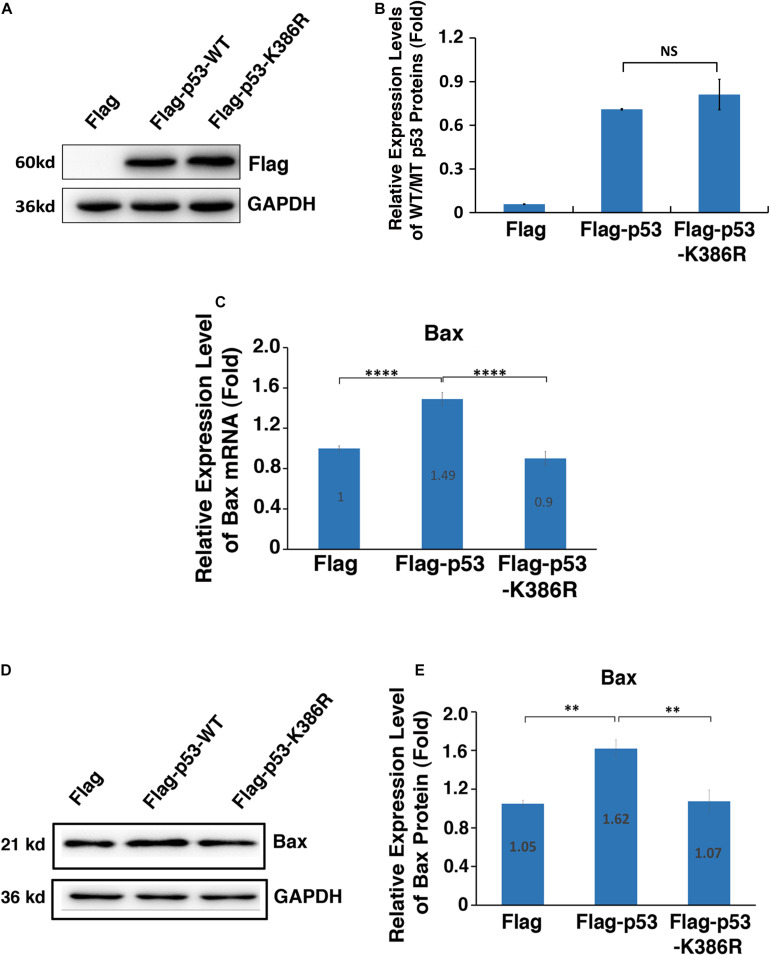
PIAS1-mediated p53 sumoylation at K386 enhances expression of the downstream proapoptotic regulator Bax. p53-KO αTN4-1 cells were transfected with Flag, Flag-p53-WT, or Flag-p53-K386R as indicated. Forty-eight hours after transfection, the mRNA or protein expression levels of exogenous p53 and Bax were analyzed. **(A)** The protein expression of exogenous p53 in three samples were analyzed by Western blot as indicated. The GAPDH was used as a loading control. **(B)** Quantification of the Western blot results in panel **(A)**. **(C)** The mRNA expression level of Bax in three samples was analyzed by qRT-PCR. Cp values of each sample were normalized with the Cp value of β-actin. **(D)** The protein expression level of Bax in three samples was analyzed by Western blot as indicated. The GAPDH was used as a loading control. **(E)** Quantification of the Western blot results in panel **(D)**. All experiments were repeated three times. Error bar represents standard deviation, *N* = 3. NS, not significant, ^∗∗^*p* < 0.01, ^*⁣*⁣**^p < 0.0001.

Since PIAS1 mediates p53 sumoylation, we next tested whether the sumoylated p53 would confer enhanced transcription activity on its downstream target genes. To do so, we transfected αTN4-1 cells with Flag vector, Flag-p53-WT, and Flag-p53-K386R plasmids separately. The endogenous p53 was knocked out in these cells (Supplementary Figure 6). As shown in Figures 6A,B, both p53-WT and p53-K386R displayed similar protein levels. Next, we examined the expression level of Bax in three different types of cells. As shown in Figure 6C, qRT-PCR revealed that overexpression of exogenous wild-type p53 enhanced 50% upregulation of Bax mRNA. Such upregulation was also detected at the protein level (Figures 6D,E). In contrast, expression of exogenous p53-K386R suppressed about 10% of Bax expression as compared with the vector Flag-transfected αTN4-1 cells with the endogenous p53 knocked out.

**FIGURE 7:**
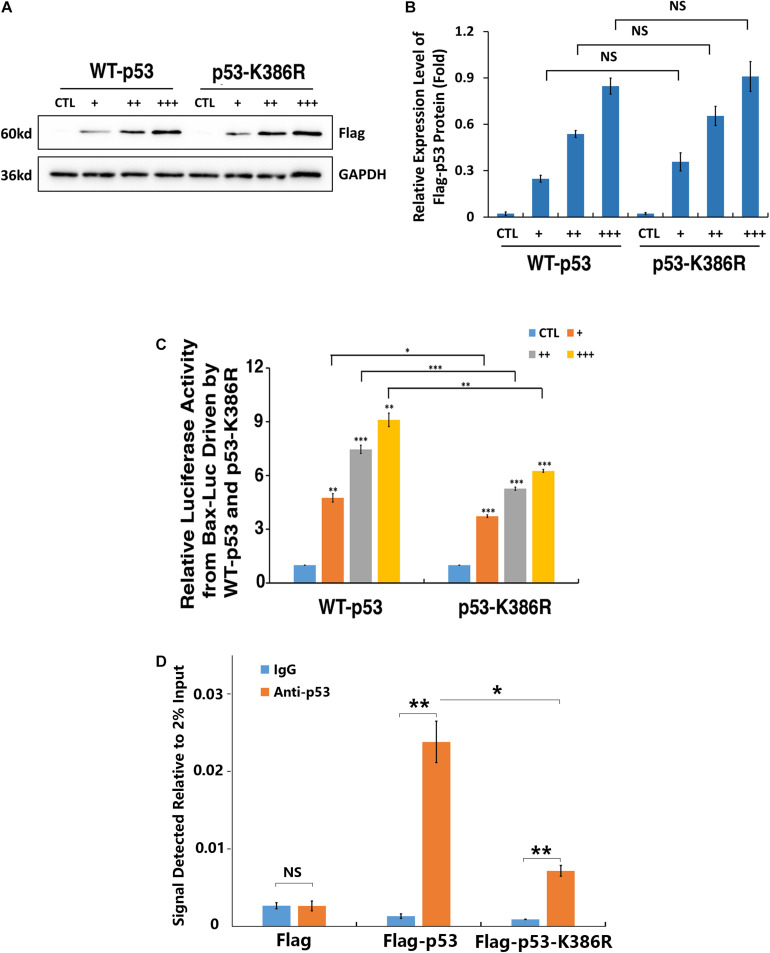
p53 sumoylation at K386 enhances the binding with Bax promoter. **(A–C)** p53-KO αTN4-1 cells were co-transfected with 1 μg of pGL3-Bax, 50 ng of pRL-TK, and empty vector (CTL), Flag-p53-WT, or Flag-p53-K386R at three different doses, 50 ng/well (+), 100 ng/well (++), and 200 ng/well (+++). **(A)** The Western blot analysis was conducted to detect exogenous p53 expression levels. The GAPDH was used as a loading control. **(B)** Quantification of the Western blot results in panel **(A)**. **(C)** Dual-luciferase activity was measured 36 h after transfection. **(D)** p53 sumoylation deficiency decreased the occupancy of p53 on the Bax promoter as measured by ChIP assay. ChIP was performed using an anti-p53 antibody and IgG as a negative control in p53-KO cells transfected with Flag, Flag-p53-WT, or Flag-p53-K386R. The occupancy was normalized to 2% DNA input and calculated in comparison with IgG control. All experiments were repeated three times. Error bar represents standard deviation, *N* = 3. NS, not significant, *p < 0.05, **p < 0.01, ***p < 0.005.

To further confirm the differential control of Bax promoter by wild-type and K386R mutant p53, we performed luciferase and ChIP assays. As shown in Figures 7A,B, the dose-dependent expression levels of WT-p53 and K386R mutant p53 with three different doses of plasmids transfected were confirmed with Western blot analysis. In the same transfected cells, significant dose-dependent upregulation of luciferase activity driven by Bax promoter was observed under overexpression of wild-type p53. Under overexpression of K386R mutant p53, however, luciferase activity from the same Bax promoter was significantly decreased with transfection of the same amount of p53 plasmids (Figure 7C). Following transfection with Flag, Flag-p53-WT, or Flag-p53-K386R in p53-KO cells, cross-linked DNA fragments were immunoprecipitated using specific anti-p53 antibody or IgG as control for ChIP assay. The resulting PCR products were quantified to determine level of p53 bound to the Bax promoter by fluorescence real-time quantitative PCR. We found that p53 level was relatively high within the Bax promoter region in wild-type p53-transfected cells but much lower when cells were transfected with p53-K386R mutant (Figure 7D). Since K386R mutant p53 was still bound to the Bax promoter with a lower affinity, we reasoned that p53 desumoylation targets the Bax promoter region to restrain Bax transcription, thus inhibiting Bax expression. Taken together, p53 sumoylation enhances expression of Bax through its direct binding to the Bax promoter region.

### Knockout of Endogenous Bax Partially Inhibited the Stress-Induced Apoptosis in Lens Epithelial Cells Expressing Exogenous PIAS1

**FIGURE 8:**
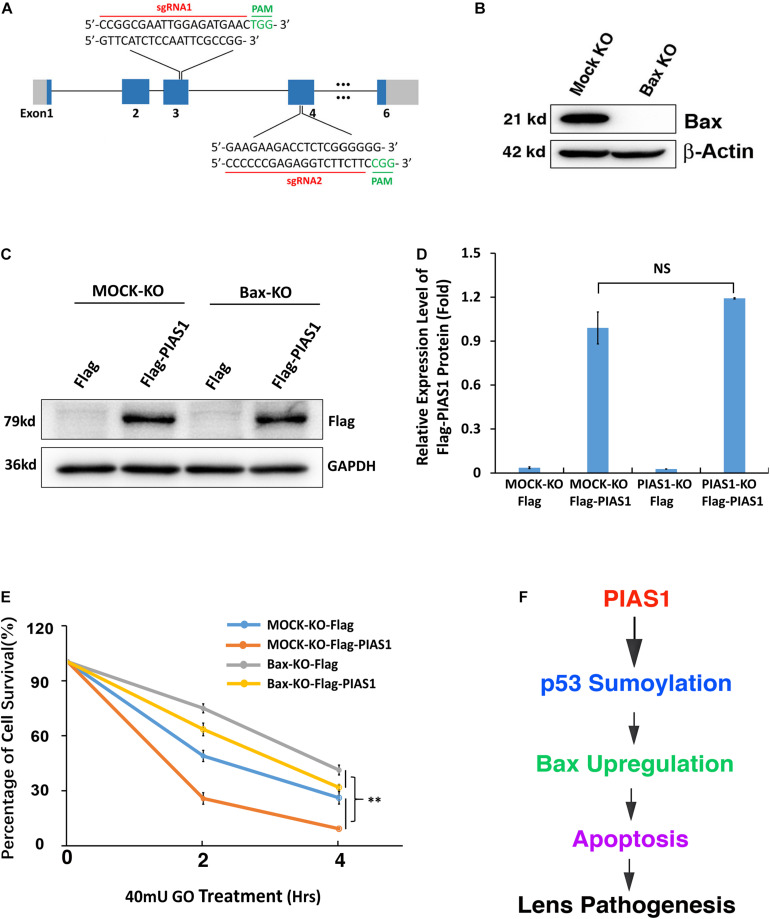
Knockout of endogenous Bax partially inhibited the stress-induced apoptosis in lens epithelial cells expressing exogenous PIAS1. **(A)** Schematic diagram of the strategy for generating the Bax knockout cells by CRISPR/Cas9 gene editing technology. **(B)** Western blot analysis of the expression levels of Bax in αTN4-1 MOCK-KO and Bax knockout (Bax-KO) cells. Note that Bax was not detectable in Bax-KO cells. The β-actin was used as a loading control. **(C)** MOCK-KO and Bax-KO cells were transfected with Flag or Flag-PIAS1 as indicated. Forty-eight hours after transfection, Western blot analysis of the expression levels of exogenous PIAS1 was conducted in MOCK-KO and Bax-KO cells. **(D)** Quantification of the Western blot results in panel **(C)**. **(E)** The transfected cells were then induced to apoptosis under 40 mU GO for 2 or 4 h and cell viability was measured by CellTiter-Glo^®^ Luminescent Cell Viability Assay. **(F)** Diagram shows how PIAS1 regulates oxidative stress-induced apoptosis of lens epithelial cells, leading to cataractogenesis. All experiments were repeated three times. Error bar represents standard deviation, *N* = 3. NS, not significant, ^∗∗^*p* < 0.01.

To further confirm that PIAS1-induced upregulation of Bax was indeed the main reason for the enhanced apoptosis of the PIAS1 overexpression cells under GO treatment, we next analyzed the effect of PIAS1 overexpression on oxidative stress-induced apoptosis in MOCK-KO and Bax-KO cells. First, we established a Bax knockout (Bax-KO) cell line using CRISPR/Cas9 technology (Figure 8A). The complete lack of Bax protein expression was verified by Western blot analysis (Figure 8B). Furthermore, the Flag vector or the Flag-PIAS1 plasmid was transfected into MOCK-KO and Bax-KO cells, and their expression in MOCK-KO and Bax-KO cells were verified (Figures 8C,D). When these cells were treated with 40 mU GO for 0, 2 and 4 h, cellTiter-Glo^®^ luminescent cell viability assays revealed that lack of Bax led to much increased levels of survival in Bax-KO cells than those in MOCK-KO cells (Figure 8E). Taken together, PIAS1 regulates stress-induced apoptosis in LECs through control of p53-sumoylation and upregulation of its target, Bax.

## Discussion

In the present study, we have demonstrated the following: (1) oxidative stress induces dose-dependent changes of both mRNA and protein of the sumoylation E3 ligase PIAS1 during oxidative stress-induced apoptosis and pathogenesis; (2) knockout of PIAS1 promotes survival during oxidative stress-induced apoptosis; in contrast, overexpression of the exogenous PIAS1 enhances stress-induced apoptosis; (3) PIAS1 overexpression upregulates expression level of the proapoptotic regulator Bax; on the other hand, PIAS1 knockout attenuates expression of Bax but shows no effect on Bak; (4) Co-IP linked Western blot analysis reveals that PIAS1 also mediates p53 sumoylation at K386 in lens epithelial cells; (5) while wild-type p53 promotes Bax expression to enhance apoptosis of lens epithelial cells, p53 K386R mutant downregulates Bax expression and thus attenuates oxidative stress-induced apoptosis; and (6) knockout of Bax significantly attenuates PIAS1 promotion of apoptosis under treatment by oxidative stress. Together, our results demonstrate that PIAS1 promotes stress-induced apoptosis, leading to the observed stress-induced cataractogenesis (Supplementary Figure 1), which is consistent with our previous studies (Li et al., 1995a,b; Li and Spector, 1996). Mechanistically, PIAS1 mediates p53 sumoylation to upregulate expression of the proapoptotic regulator Bax. Thus, our study provides novel evidence to show that sumoylation is linked to lens cataractogenesis (Figure 8F).

### Protein Sumoylation Regulates Both Ocular Development and Pathogenesis

During ocular development, previous studies from different laboratories including ours have demonstrated that sumoylation plays critical roles in retina differentiation (Onishi et al., 2010; Roger et al., 2010) and lens formation (Yan et al., 2010; Gong et al., 2014). Sumoylation determines the differentiation direction of cone and rod cells in the retina (Onishi et al., 2010; Roger et al., 2010). SUMO1-conjugated sumoylation is necessary to activate the function of the p32 Pax6 (Yan et al., 2010), which plays a fundamental role in establishing the lens placode and vesicle (Cvekl and Ashery-Padan, 2014). Moreover, we demonstrated that different SUMOs can act on the same transcription factor to display contrast functions in regulating lens differentiation (Gong et al., 2014). Since sumoylation provides an indispensable function in ocular development, we speculate that sumoylation may also be implicated in ocular pathogenesis, especially during stress-induced cataract formation.

Cataract is defined as the opacity in the ocular lens, which is derived from genetic mutations (Shiels and Hejtmancik, 2019), environmental stresses, and aging (Spector, 1995; Li, 1997). At the cellular level, our previous studies have shown that various stress conditions such as oxidative stress (Li et al., 1995a), UV irradiation (Li and Spector, 1996), and abnormal calcium (Li et al., 1995b) can all induce apoptosis of lens epithelial cells followed by cataractogenesis. At the molecular level, our recent study of the sumoylation patterns in different age groups of cataract patients revealed that sumoylation of total proteins in the capsular epithelial cells of cataractous lenses are significantly enhanced from 50 to 70 s (Liu et al., 2020), indicating that sumoylation is linked to cataractogenesis. In the present study, we demonstrated that during oxidative stress-induced cataractogenesis, the sumoylation E3 ligase PIAS1 is upregulated in the first 120 min (Figure 1). The upregulated PIAS1 is capable to promote p53 sumoylation at K386 residue (Figure 3 and Supplementary Figure 4). As a result, expression of the p53 downstream target Bax becomes upregulated, which triggers stress-induced apoptosis (Figures 5**–**8), leading to observed cataractogenesis (Supplementary Figure 1 and Figure 8F). Thus, our results further confirm that sumoylation promotes cataractogenesis.

In retina, we have recently revealed that during oxidative stress-induced age-related macular degeneration (AMD), de-sumoylation of p53 is essential to mediate heterochromatin protection of retinal pigmental epithelial cells under the oxidative stress insult (Gong et al., 2018). Taken together, sumoylation regulates both ocular development and pathogenesis.

### PIAS1 Regulates Different Targets to Promote or Attenuate Apoptosis

The proapoptotic function of PIAS1 was initially demonstrated in human 293T cells and human osteosarcoma U2OS cells. It was found that ectopic expression of PIAS1 in U2OS cells activated JNK1 and triggered apoptosis (Liu and Shuai, 2001). Subsequently, in H1299 cells, it was found that PIAS1 can activate p53-mediated gene expression such as p21 independent of its ligase activity (Megidish et al., 2002). More recently, Yang et al. (2013) found that exposure to zinc induced apoptosis of prostate cancer cells and resulted in transactivation of p21 (WAF1/Cip1) in a Smad-dependent and p53-independent manner. During this process, it was found that PIAS1-modulated Smad2/4 complex activation plays an important role (Yang et al., 2013).

In the present study, we did not observe PIAS1 activation of JNK under PIAS1 overexpression or knockout (Supplementary Figure 3). Therefore, we sought after other mechanisms mediating PIAS1 promotion of apoptosis. Since the tumor suppressor p53 is a master regulator of apoptosis (Kruiswijk et al., 2015), we next considered if PIAS1 regulates p53.

Previous studies have shown that p53 is SUMO-1 conjugated at K386 residue (Gostissa et al., 1999; Rodriguez et al., 1999; Muller et al., 2000; Kahyo et al., 2001; Kwek et al., 2001; Schmidt and Muller, 2002; Okubo et al., 2004). Moreover, regarding the ligase mediating p53 sumoylation, Kahyo et al. (2001) revealed that PIAS1 can interact with p53 and catalyze its sumoylation in U2OS cells (Kahyo et al., 2001). In the present study, we confirmed the results of these earlier studies in mouse lens epithelial cells. *In vitro* mutagenesis and Co-IP linked Western blot analysis revealed that PIAS1 mediates p53 sumoylation at K386 residue (Figure 3 and Supplementary Figure 4). Furthermore, we demonstrated that sumoylated p53 promotes expression of its downstream proapoptotic regulator, Bax (Figures 6, 7). Bax upregulation plays a crucial role in mediating PIAS1 induction of apoptosis of lens epithelial cells under stress condition since Bax knockout through CRISPR/Cas9 technology significantly attenuates PIAS1-mediated apoptosis (Figure 8). Together, here we demonstrate that Bax but not Bak is a novel key target to mediate PIAS1-promoted apoptosis in mouse lens epithelial cells.

Besides its role to promote apoptosis, PIAS1 can modulate different targets to promote survival. AKT is an important kinase to promote survival (Xiao et al., 2010). PIAS1-mediated AKT sumoylation at K276 by SUMO1 is crucial for its activation. Both K276R and E278A mutations reduce AKT sumoylation, completely abolish AKT kinase activity, but do not affect its ubiquitination (Li et al., 2013). More recently, Xie et al. (2018) demonstrated that PIAS1 can protect against myocardial ischemia–reperfusion injury by stimulating PPARγ sumoylation (Xie et al., 2018). Thus, PIAS1 can promote both apoptosis and survival depending on environmental conditions, tissue specificity, and the targets modified.

In summary, our present study confirms that sumoylation plays an important role in lens pathogenesis. Specifically, we demonstrate that PIAS1, an important E3 ligase for sumoylation, is subjected to regulation by oxidative stress. Oxidative stress-induced PIAS1 expression promotes p53 sumoylation and, therefore, upregulates expression of the proapoptotic regulator Bax. Together, we have shown that Bax is a novel target to mediate PIAS1 induction of apoptosis. PIAS1 promotes oxidative stress-induced apoptosis through the p53-Bax pathway, leading to cataractogenesis.

## Materials and Methods

### Materials

Various molecular biology reagents were purchased from MP Biomedicals Ltd., CA, and Invitrogen Life Technologies, Gaithersburg, MD. All the oligos were purchased from Sangon Biotech (Shanghai) Co., Ltd. Protein size markers were purchased from GenStar (Beijing) Co., Ltd. Various antibodies were obtained from Cell Signaling Technology, Boston, MA; abCam Inc., Cambridge, MA; Santa Cruz Biotechnology, Inc., Dallas, TX; Sigma-Aldrich, St. Louis, MO; Proteintech (Wuhan) Co., Ltd; and Ray Biotech (Beijing) Co., Ltd.

### Animals

Four-week-old C57BL/6J mice were used to induce an *in vitro* cataract model (Supplementary Figure 1). Mice were housed in standard cages in a specific pathogen-free facility of Sun Yat-sen University. The room was maintained on a 12-h light–dark cycle at a constant temperature of 25°C and 50% humidity and the animals were fed with commercial laboratory food and sterilized water. In all the cases, animal protocols to use mice were approved by the IACUC of Zhongshan Ophthalmic Center of Sun Yat-sen University.

### Cell Culture, Plasmid Construction, and Establishment of Gene Overexpression or Knockout Stable Cell Lines

Mouse lens epithelial cell line αTN4-1 was cultured in Dulbecco’s modified Eagle’s medium (DMEM) supplemented with 10% fetal bovine serum (FBS; Atlanta Biologicals) and 1% penicillin/streptomycin in 5% CO_2_ at 37°C.

The CRISPR/Cas9-based gene KO vector pSp Cas9(BB)-2A-Puro (PX459) is a gift from Dr. Mengqing Xiang in Zhongshan Ophthalmic Center of Sun Yat-sen University. The sgRNA sequences used for PIAS1, p53, and Bax gene KO are shown in Supplementary Table 1. These sgRNAs were inserted into PX459 using the *Bbs*I restriction sites. For expression vector, the construction of full-length cDNA of PIAS1 or p53 was cloned by PCR. The PIAS1 cDNA was first subcloned into an enhanced green fluorescence protein expression vector, pEGFP-C3, at the *Eco*RI and *Bam*HI sites, and subsequently was subcloned into a Flag expression vector, p3 × FLAG-CMV-10, at the *Hin*dIII and *Kpn*I sites. The p53 cDNA was subcloned into a Flag expression vector, p3 × FLAG-CMV-10, at the *Eco*RI and *Xba*I sites. The point mutation of p53 (K386R) and PIAS1(C351S) primers were designed according to the QuikChange Site-Directed Mutagenesis. The primers used for gene overexpression and point mutation are listed in Supplementary Table 1.

For establishment of stable cell lines, αTN4-1 cells were transfected with the above plasmids using Lipofectamine 3000 (Life Technologies) according to the manufacturer’s instructions. Forty-eight hours after transfection, cells stably expressing the plasmids were selected by 1.0 μg/ml puromycin for PX459 and 500 μg/ml G418 for pEGFP-C3 and p3 × FLAG-CMV-10. After about 4 weeks, individual clones for the stable cell lines were established and confirmed by Western blot analysis and DNA sequencing.

### Protein Extraction and Western Blot

Total proteins were extracted by RIPA buffer (1% NP-40, 1% sodium deoxycholate, 0.1% SDS, 50 mM Tris-HCl, pH 8.0, and 150 mM NaCl) supplemented with the Protease Inhibitor Cocktail and then cell lysates were sonicated and centrifuged at 13,000 rpm for 10 min at 4°C. The supernatants were transferred to new tubes. Fifty micrograms of total proteins in each sample were separated by 10 or 12% SDS-polyacrylamide gel and transferred to PVDF membranes. The protein blots were blocked with 5% non-fat milk in TBST (10 mM Tris-HCl, pH 8.0, 150 mM NaCl, and 0.05% Tween-20) and further incubated with primary antibodies overnight at 4°C. Primary antibodies used in Western blot are shown in Supplementary Table 2. The horseradish peroxidase-conjugated secondary antibodies (CST; #7077 and #7074) were then applied for 1 h at room temperature. Immunoreactivity was detected with a chemiluminescence detection kit (ECL Ultra; New cell & Molecular Biotech Co., Ltd.), and the blots were visualized using a Tanon chemiluminescence system (China).

### Free Thiol Content Assay

The Free Thiol Content Assay was performed as previously described (Sun et al., 2020; Wang et al., 2020) and using the fluorometric thiol quantitation kit (Sigma-Aldrich, #MAK151). Briefly, αTN4-1 cells were treated with GO and then washed with PBS three times, lysed in 150 μl of the assay buffer, and 20 μl of the cell lysates were used for each assay reaction. The reaction was allowed for 10–60 min at room temperature in the dark (with foil to wrap the plate). The fluorescence intensity was measured by Synergy H4 Hybrid Microplate Reader (BioTek, Winooski, VT, United States) at 490 nm (ex) and 535 nm (em).

### Apoptosis Assays

Cell apoptosis was determined by cellTiter-Glo^®^ luminescent cell viability assay, flow cytometry, and Live/Dead Viability/Cytotoxicity. The cellTiter-Glo ^®^ luminescent cell viability assay was conducted as previously described (Wang et al., 2020). The flow cytometry was performed with the PE Annexin V Apoptosis Detection Kit I (559763; BD Pharmingen). Cells were seeded into six-well plates about 70% confluence and then treated with 40 mU GO for different times as indicated in the figures to induce cell apoptosis. After stimulation, cells were collected and washed with cold PBS, finally suspended in binding buffer, and 5 μl each of annexin V (PE) and 7-amino-actinomycin (7-AAD) were added into suspended cells and incubated at room temperature for 20 min. The stained cells were analyzed with a BD LSRFortessa Cell analyzer (649225; BD). The Live/Dead Viability/Cytotoxicity Kit (L3224; Thermo Fisher Scientific) was used to distinguish live and dead cells. Live cells are characterized by the presence of ubiquitous intracellular esterase activity and revealed by calcein AM. The polyanionic dye calcein is well retained within live cells, producing an intense uniform green fluorescence in live cells. EthD-1 enters cells with damaged membranes and displays red fluorescence upon binding to nucleic acids, thereby producing a bright red color in dead cells and EthD-1 is excluded by the membrane of live cells. The images were captured with a Zeiss microscope.

### Immunoprecipitation

Whole-cell extracts were prepared with lysis buffer (25 mM Tris-HCl, pH 7.4, 150 mM NaCl, 5% glycerol, 1% Non-idetP-40, and 1 mM EDTA) and precleared with Protein A/G PLUS-agarose beads (sc-2003; Santa Cruz Biotechnology). Pre-cleared lysates were then incubated with anti-p53 antibody (sc-126; Santa Cruz Biotechnology and #2524, CST) or anti-FLAG antibody (F1804; Sigma-Aldrich) overnight, followed by incubation with Protein A/G PLUS-agarose beads for 2 h at 4°C. The eluted proteins were analyzed by Western blots. For detection of sumoylated p53, freshly prepared 20 mM SENP inhibitor, NEM, was added during cell lysis and washing steps.

### RNA Extraction and qRT-PCR

Total RNAs from αTN4-1 cells were isolated using TRIzol Reagent (Invitrogen). One microgram of total RNA was transcribed (RT) into 20 μl of cDNA using the HiScript II Q RT SuperMix for qPCR kit (R223-01; Vazyme) in which the genomic DNA was removed by DNase I digestion. Fluorescence real-time quantitative PCR was performed on the LightCycler 480 qPCR system (Roche) with ChamQ SYBR Color qPCR Master Mix (Q411-02; Vazyme) according to the manufacturer’s procedures. The assays were performed in triplicate, and the Ct values were normalized to β-actin. The primers used are listed in Supplementary Table 1.

### Luciferase Assay

The DNA fragment containing mouse Bax promoter region was amplified by PCR using the primers listed in Supplementary Table 1. Next, the fragment was cloned into the pGL3 promoter vector containing a luciferase reporter gene at the *Kpn*I and *Hin*dIII sites. Renilla luciferase pRL-TK (Promega) vector was used to normalize the background in these experiments. For our luciferase assays, approximately 80% confluent p53-KO αTN4-1 cells were co-transfected with 1 μg of pGL3-Bax, 50 ng of pRL-TK, and varied amounts (50, 100, or 200 ng/well) of empty vector (CTL) or Flag-p53, Flag-p53-K386R, and then incubated for 36 h. The Dual-Luciferase Reporter Assay System (#RG027, Beyotime) was used to quantify luminescence from transfected cells, and the Firefly and Renilla luciferase signals were measured by the Synergy H4 Hybrid Microplate Reader (BioTek, Winooski, United States). Relative luciferase activity (Luc) was calculated by the ratio of Firefly and Renilla luciferase signals.

### ChIP Assay

The ChIP assay was performed using the ChIP chromatin immunoprecipitation kit (CST, 9003) according to the manufacturer’s instruction. P53 KO cells were transfected with Flag, Flag-p53-WT, or Flag-p53-K386R. Forty-eight hours later, the transfected cells were fixed in 20 ml of DMEM with 1% formaldehyde at room temperature for 10 min. The cells were washed with PBS, and the harvested cells were sonicated 45 times for 2 s each at power 60% to produce chromatin DNA fragments between 150 and 900 bp in size. Supernatants were collected for IP with 5 μg of p53 antibody (CST; #2524) or 5 μg of normal IgG through overnight incubation at 4°C. Next, 30 μl of magnetic protein A/G beads was added into the mixtures and allowed incubation for 2 h at 4°C. Protein–DNA complexes were washed with low salt buffer three times and high salt buffer one time, and eluted from magnetic protein A/G beads with elution buffer at 65°C for 30 min. Crosslinks were reversed by adding 6 μl of 5 M NaCl and 2 μl of proteinase K into the mixture, and the reaction mixture was incubated for an additional 2 h at 65°C. Finally, DNA fragments were purified on Spin columns and subsequently used for qPCR. The primers used for ChIP-qPCR are listed in Supplementary Table 1.

### Statistics

All results shown are reported as the mean ± standard deviation (SD). Significance was calculated using the unpaired two-tailed *t*-test. Differences were considered statistically significant at *p* < 0.05.

## Data Availability Statement

The raw data supporting the conclusions of this article will be made available by the authors, without undue reservation.

## Ethics Statement

The animal study was reviewed and approved by The IACUC of Zhongshan Ophthalmic Center of Sun Yat-sen University.

## Author Contributions

QN and DWL designed the research and wrote the manuscript. QN, HC, MZ, LW, MH, J-WX, ZL, X-DG, J-LF, YW, S-YZ, YX, Y-WG, QG, Y-YB, and J-MW performed the experiments. QN, LZ, X-CT, XH, LG, YL, and DWL analyzed the data. All authors contributed to the article and approved the submitted version.

## Conflict of Interest

The authors declare that the research was conducted in the absence of any commercial or financial relationships that could be construed as a potential conflict of interest.
