# Supplementary material for: The E3 Ligase PIAS1 Regulates p53 Sumoylation to Control Stress-Induced Apoptosis of Lens Epithelial Cells Through the Proapoptotic Regulator Bax
Source: Front Cell Dev Biol. 2021 Jun 14;9:660494. doi: 10.3389/fcell.2021.660494 (PMC8237824; doi:10.3389/fcell.2021.660494)
Supplement: Supplementary file 1 [file Data_Sheet_1.pdf]

Supplementary Data

1) **Supplementary Figures**

Fig. S1 Nie et al.

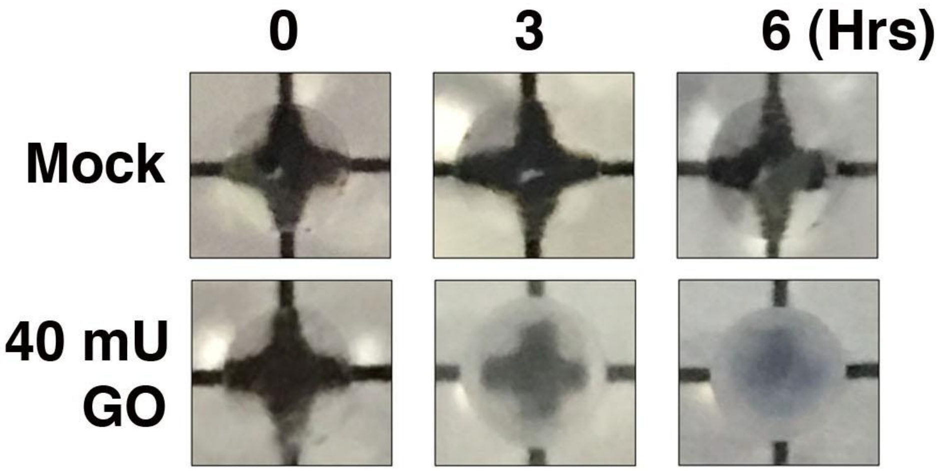

Figure S1. Glucose oxidase (GO) treatment-induced in vitro cataract model.

Lenses from four-week-old C57BL/6J mice were carefully excised from eyes and cultured in M199 medium (Sigma 3769) for at least 12 hours. The transparent lenses were then treated with 40 mU GO to induce cataract formation. Photographs of lenses with mock treatment (top) or 40 mU GO treatment for 0h, 3h and 6h (bottom). Note that after GO treatment, the transparent lens became opaque at the cortical area at 3-hour and almost complete opaque at 6-hour.

Fig. S2 **Nie et al.**

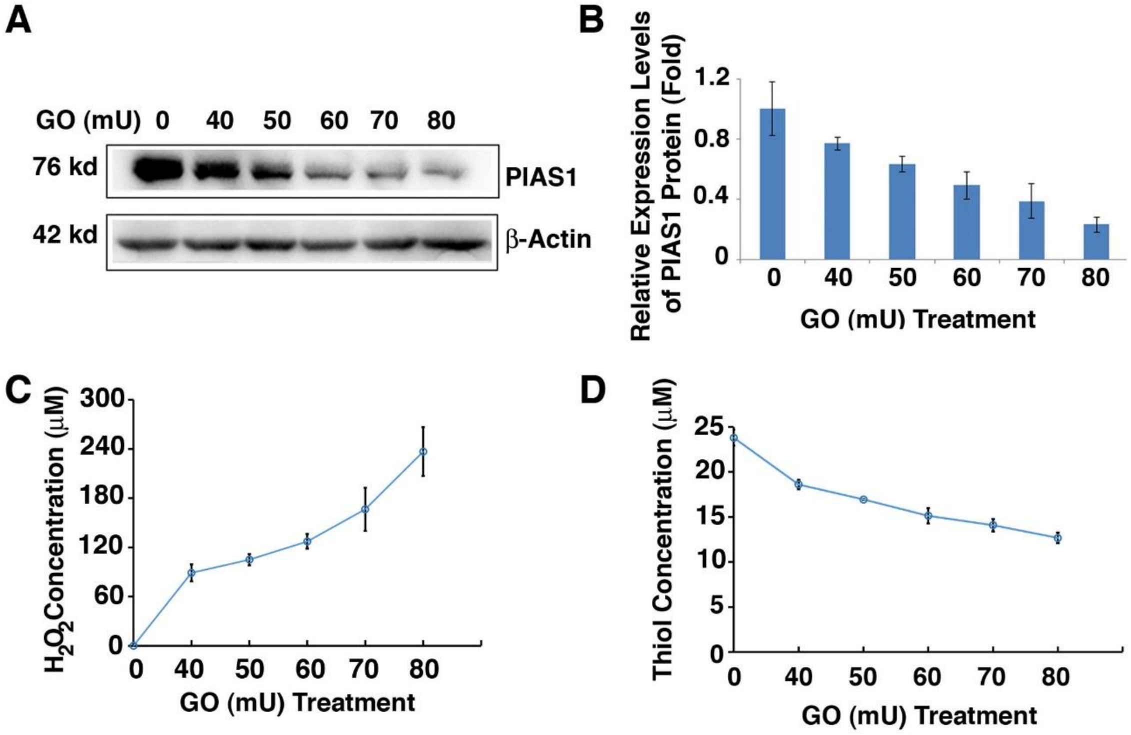

Figure S2. Oxidative stress induces dose-dependent PIAS1 downregulation under different amounts of GO treatment in lens epithelial cells.

(A) Western blot analysis showed the dose-dependent decrease in protein expression level of PIAS1 with different amounts of GO treatment as indicated for three hours. (B) Quantification of the western blot results in Figure S2A. (C) Dynamic H_2O_2 concentration generated from 0 to 80 mU GO in αTN4-1 cells for three hours. (D) Dynamic changes of free thiol content upon 0 to 80 mU GO treatment in αTN4-1 cells for three hours. All experiments were repeated three times. Error bar represents standard deviation, N=3.

Fig. S3 Nie et al.

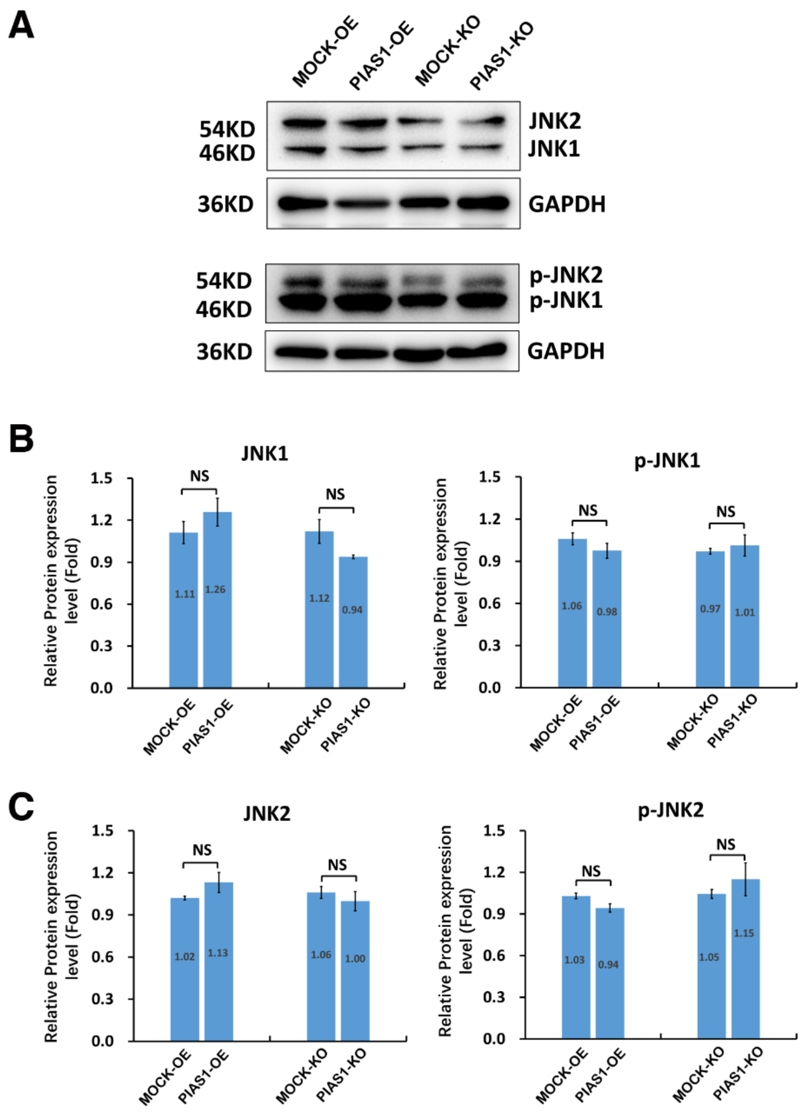

Figure S3. Changes of PIAS1 levels have no effect on the expression and activation of JNK in αTN4-1 cells.

(A) Western blot analysis of the protein expression levels of JNK and p-JNK in MOCK OE and PIAS1-OE, MOCK-KO and PIAS1-KO cells. The GAPDH was used as a loading control. (B-C) Quantification of the western blot results in Figure S3A. NS, not significant.

Fig. S4 **Nie et al.**

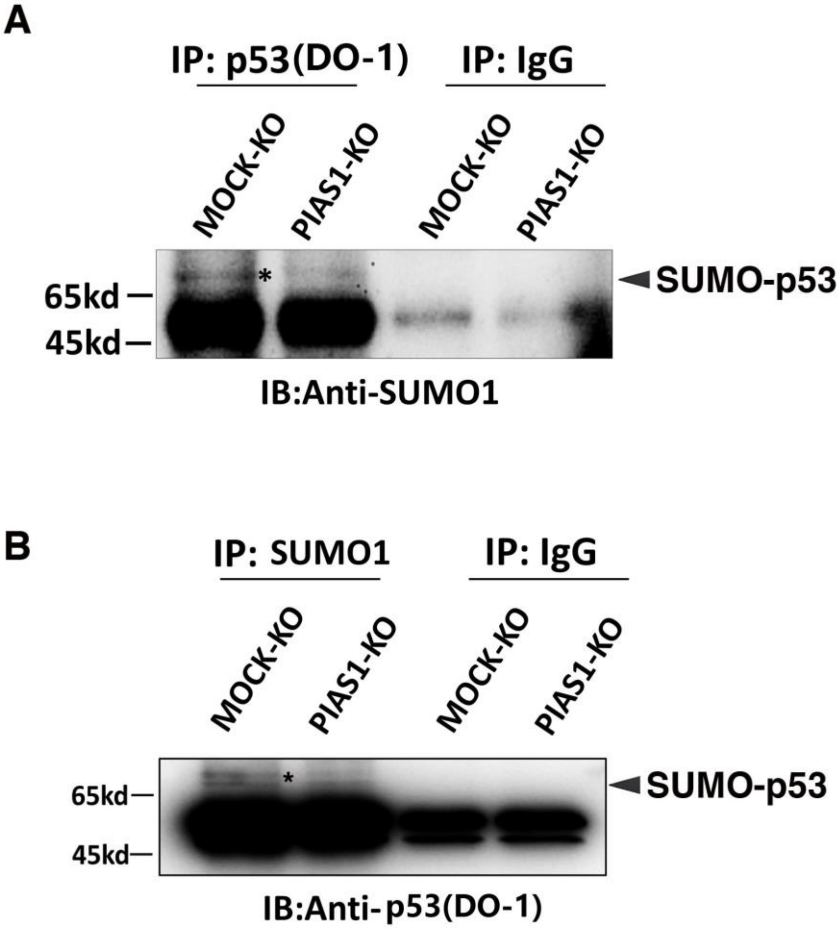

Figure S4. Sumoylation of endogenous p53 was mediated by PIAS1 in αTN4-1cells.

The total proteins from the MOCK-KO and PIAS1-KO αTN4-1 cells were extracted with IP lysis buffer. (A) The extracted total proteins were then immunoprecipitated with anti-p53(DO-1) antibody followed by SUMO1 immunoblotting. (B) The extracted total proteins were then immunoprecipitated with anti-SUMO1 antibody followed by p53(DO-1) immunoblotting. The sumoylated p53 was labeled with*.

Fig. S5 Nie et al.

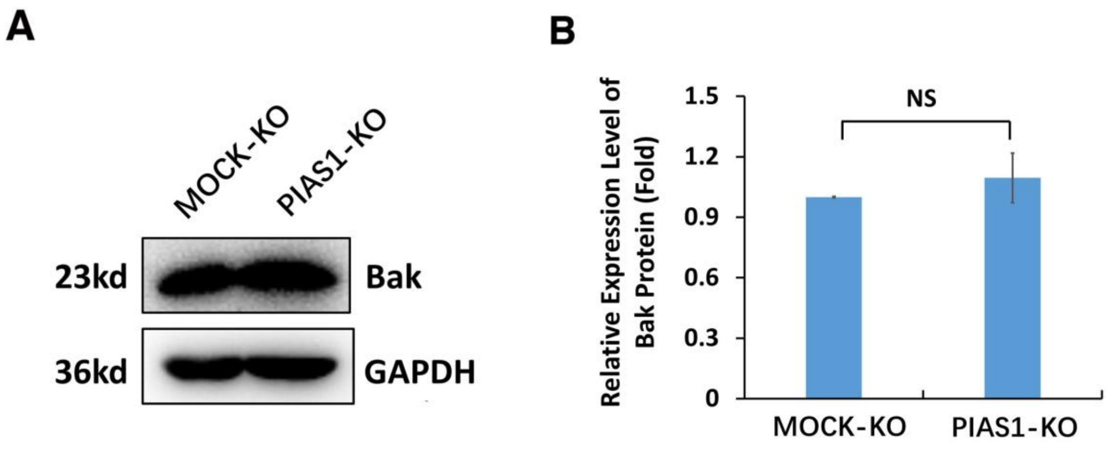

Figure S5. Western blot result of Bak expression, another member of Bcl-2 family in αTN4-1-MOCK-KO and αTN4-1-PIAS1-KO cells.

(A) Western blot analysis of the expression level of Bak in αTN4-1-MOCK-KO and αTN4-1-PIAS1-KO cells. The GAPDH was used as a loading control. (B) Quantification of the western blot results in Figure S5A. NS, not significant.

Fig. S6 Nie et al.

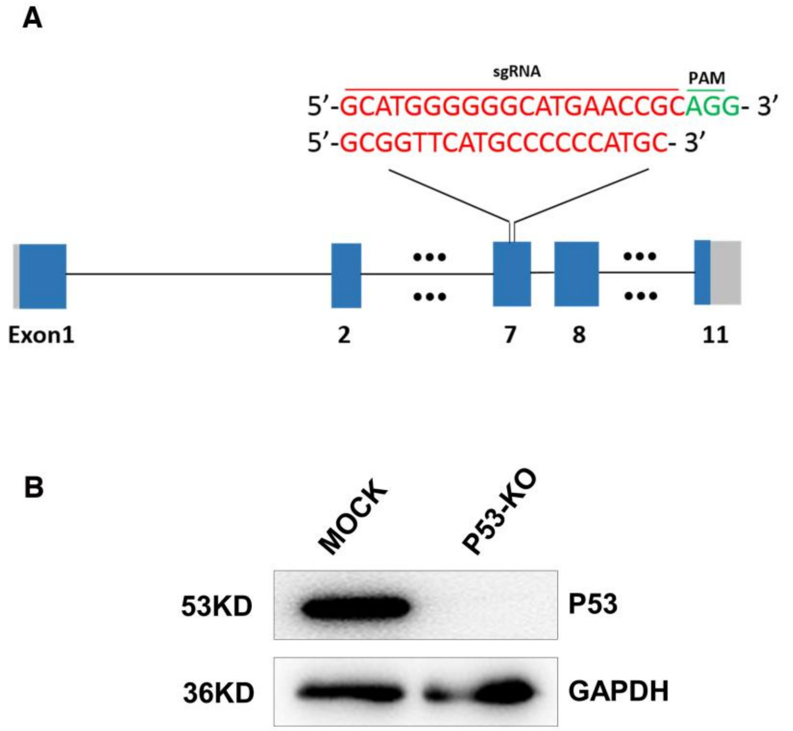

Figure S6. Generation and characterization of p53 knockout αTN4-1 cells.

(A) Schematic diagram of the strategy for generating the p53 knockout cells by CRISPR/Cas9 gene editing technology. Two sets of sgRNAs were used to generate the knockout cell line. (B) Western blot analysis of the expression levels of p53 in MOCK-KO and p53 knockout (p53-KO) cells. Note that p53 was not detectable in p53-KO cells. The GAPDH was used as a loading control.

2) Supplementary Tables

Table S1. Primers used in gene knockout, overexpression, point mutation, qRT-PCR, Luciferase assay and ChIP-qPCR assay.

Primer Name	Primer direction	Primer sequences
Mouse-PIAS1 (qRT-PCR)	F	CAGTGCGGAACTAAAGCAAATGG
	R	GGAACCGCCTCCTGTAGAGT
Mouse PIAS1 sgRNA (Knockout)	F	CACCGAACAGGTTTCCCGAAAGCGC
	R	AAACGCGCTTTCGGGAAACCTGTTC
Mouse p53 sgRNA (Knockout)	F	CACCGCGGTTCATGCCCCCCATGC
	R	AAACGCATGGGGGGCATGAACCGC
Mouse Bax sgRNA1 (Knockout)	F	CCGGCGAATTGGAGATGAACTGG
	R	CCAGTTCATCTCCAATTCGCCGG
Mouse Bax sgRNA2 （Knockout）	F	CCCCCCGAGAGGTCTTCTTC
	R	GAAGAAGACCTCTCGGGGGG
Mouse PIAS1 in pEGFP (Overexpression)	F	CGGAATTCTGATGGCGGACAGTGCGGAA
	R	CGGGATCCTCAGTCCAATGAGATAAT
Mouse PIAS1 in p3×FLAG (Overexpression)	F	CGCAAGCTTATGGCGGACAGTGCGGAACTAAA
	R	ATGGGTACCTCAGTCCAATGAGATAATGTCTGGTATGATGCCA
Mouse PIAS1 C351S (Point Mutation)	F	CTGAAGGTGGGAGGAGGTAAGTGCCCG
	R	CGGGCACTTACCTCCTCCCACCTTCAG
Mouse p53 in p3×HA (Overexpression)	F	CGGAATTCATGACTGCCATGGAGGAGT
	R	GCTCTAGATGGGGAACAAGAAGTGGAG
Mouse-Bax (qRT-PCR)	F	AGGATGCGTCCACCAAG
	R	AAAGTAGAAGAGGGCAACCA
Mouse-β-actin (qRT-PCR)	F	TAGGCACCAGGGTGTGATGG
	R	CTCCATGTCGTCCCAGTTGGT
Mouse-Bax-Promoter (Luciferase Assay)	F	GGGGTACCGCTTTGGGCTCGGGCGGGAG
	R	CCCAAGCTTAAGCAAACAGACCCCCAGCT
Mouse-Bax (ChIP-qPCR)	F	AGAGGCAGCGGCAGTGAT
	R	GCTCCACAGCAAACCCAGA

Table S2. Primary antibodies used in Western blot, Co-IP and ChIP assays.

Primary Antibody	Cat. Number
PIAS1	Cell Signaling Technology ; 3550
GFP	Beijing Ray Biotech ; RM1008
p53 (DO-1)	Santa Cruz Biotechnology ; sc-126
p53	Cell Signaling Technology ; 2524
SUMO1	Sigma-Aldrich ; 8070
Flag	Sigma-Aldrich ; F1804
Bax	abCam ; ab32503
β-actin	Proteintech ; 66009
GAPFH	Proteintech ; 60004
JNK	Cell Signaling Technology ; 9252
p-JNK	Santa Cruz Biotechnology ; sc-6254
Bak	Cell Signaling Technology ; 12105
